# Use of Steel Industry Wastes for the Preparation of Self-Cleaning Mortars

**DOI:** 10.3390/ma12040621

**Published:** 2019-02-19

**Authors:** José Balbuena, Luis Sánchez, Manuel Cruz-Yusta

**Affiliations:** Univ Cordoba, Inst Univ Invest Quim Fina & Nanoquim IUNAN, Dept Quim Inorg, Campus Rabanales, Edif. Marie Curie 1ª Planta, E-14071 Cordoba, Spain; donjosebj@gmail.com (J.B.); luis-sanchez@uco.es (L.S.)

**Keywords:** waste, iron oxide, photocatalytic, self-cleaning

## Abstract

An important problem, which must be solved, is the accumulation of industrial waste in landfills. Science has an obligation to transform this waste into new products and, if possible, with high added value. In this sense, we propose the valorization of the waste which is generated in the steel lamination process (HSL) through its conversion into a new material with photocatalytic activity which is suitable for use as an additive to obtain a self-cleaning construction material. The valorization of steel husk lamination waste is achieved through a grinding process, which allows the sample to be homogenized, in size, without altering its phase composition, and a thermal treatment that turns it into iron oxide, which acts as a photocatalyst. These residues, before and after treatment, were characterized by different techniques such as PXRD (Powder X-Ray Diffraction), TGA (Thermogravimetric Analysis), SBET (Specific surface area, Brunauer-Emmett-Teller), SEM (Scanning Electron Microscopy) and Diffuse reflectance (DR). MB and RhB tests show that this material is capable of self-cleaning, both of the material itself and when it is incorporated into a construction material (mortar). In addition, the NO_x_ gas elimination test shows that it is also capable of acting on greenhouse gases such as NO_x_.

## 1. Introduction

Every day, society demands more responsible usage of natural resources and reduced generation of waste. Green chemistry is defined as “the effort of reducing or eliminating the use or generation of hazardous substances in the design, manufacture and application of chemical products” [1]. In this context, green chemistry would be a tool for society, eliminating or reducing the use of chemical reagents which are harmful to the environment in the design and manufacture of new materials. Using green chemistry, much of the waste generated in industrial processes can become co-products, thereby reducing the consumption of natural resources.

Green chemistry has numerous tools, one of them being photochemistry. The use of light as a chemical reagent induces chemical changes that enable the elimination of some environmental pollutants.

Proper treatment of waste can lead to new co-products, but if we also use our imagination, we can make co-products with high added value. For example, in the case of photocatalysis, most investigations are based on the use of TiO_2_ as the semiconductor material; however, photochemical reactions could be carried out with other semiconductors such as Fe_2_O_3_, ZnO, etc. With proper treatment, waste containing any of these compounds, or a mixture thereof, could be converted into new products for advanced applications [2,3].

To achieve an optimum recovery of a given type of waste, it is important to know the sector in which the material is intended to be reintroduced, as the requirements are different when reusing liquid or solid wastes [4,5]. The construction industry is able to accept different types of wastes which, if properly treated, can reduce the consumption of large amounts of raw materials [6]. Therefore, by managing new efforts, green chemistry could lead to the development of new advanced building materials. In this sense, the use of sustainable processes could be suitable for solving today’s environmental problems such as aesthetic and air pollution in big cities, mainly caused by the road traffic [7].

Due to the presence of aerosol pollutants, the appearance of visible stains on the surface of buildings is common. Thus, pollution remains on the facades of buildings, with hydrocarbons facilitating adhesion onto the surface [3]. Air pollution is due, among others factors, to the presence of nitrogen oxides. Modern society is concerned about pollution because of its harmful effects on human health. Usually, photochemical processes are proposed in order to cut down the presence of such gases in the atmosphere, with TiO_2_ being the main photocatalyst used. It possible to carry out photocatalytic oxidation (PCO) of NO_x_ gases with building materials that contain a semiconductor, usually TiO_2_. This PCO is possible thanks to atmospheric oxygen, humidity and solar radiation [8,9,10,11]. In the case of the latter, when exposed to sunlight, TiO_2_ also initiates the photochemical decomposition of organic dust deposited on building surfaces [12,13]. However, TiO_2_ as an additive in the formulation of a construction material causes an important increase in the final cost of these products, so that the large-scale use of these materials is still quite unfeasible.

Consequently, it is of interest to find alternative low-cost photocatalytic additives. Under atmospheric conditions, the most stable semiconducting iron oxide is hematite (α-Fe_2_O_3_). Although it has less photocatalytic activity than TiO_2_, it has the advantage of absorbing it in the region of visible light (1.9−2.2 eV band gap), and this makes it especially interesting for the photocatalytic removal of contaminants in both water and air [14,15]. The iron oxides can be obtained from waste enriched in iron and, through proper treatment, the corresponding photocatalyst is obtained, as previously reported by our research group [2,16].

In this sense, an interesting resource is waste from the steel industry. Each year the steelmaking process generates approximately 3 million tons of waste oxides. Suppressing the need to send these wastes to landfill will save the industry approximately $120,000,000. Large amounts of this type of waste are produced during the steel lamination process. Because of its high iron oxide content, this waste is suitable for the preparation of raw materials with photocatalytic properties.

The present work illustrates an initial study on the valorization of waste generated during the husk of steel lamination process (HSL). The proper treatment of this waste, herein described, allows its transformation into an added-value product, which is used as a photocatalytic additive in the preparation of new self-cleaning and self-depolluting materials. Despite the number and variety of waste generated in the steel industry and the many reports on the valorization of this type of waste [17,18], only two previous works have been devoted to its transformation as a photocatalyst [19,20]. In both cases, the composition of the waste and the valorization process were very different. 

As a novelty in the present work, we have prepared new co-products made from waste from the steel lamination process with new qualities, such as photocatalytic properties, and have evaluated their self-cleaning capacity and air purification capacity to remove greenhouse gases such as NO.

## 2. Materials and Methods 

### 2.1. Materials

The solid waste generated as husk from the steel lamination process (HSL) was the raw material we evaluated in this study. It was provided by a national waste management company (FCC Ámbito, Córdoba, Spain). Many waste samples, received from the provider, were studied, the main feature being the uniformity in composition. The received sample was calcined to 400 °C until constant weight was achieved to remove all traces of oils and derivatives present in the sample; Figure S1. This pre-conditioned sample was denoted as HSL. To homogenize the particle size, 50 g of the sample was ground using a planetary mill Retsch PM100 (Retsch, Haan, Germany) with a stainless steel 250 mL vessel and six balls, for 1 h at 300 rpm. To obtain the photocatalityc active iron phases, α-Fe_2_O_3_ (hematite phase), the sample was thermally and mechanically treated. This HSL calcined sample is an iron rich waste and could be potentially be used as a photocatalyst. X-ray fluorescence analysis (XRF; Philips PW2404 X-ray spectrometer, Malvern Panalytical, Royston, UK) was used to determine the chemical composition. In order to determine the crystalline phases of the raw materials, X-ray diffraction analysis (XRD; Siemens D5000 X-ray diffractometer with CuKα_1,2_ radiation, Siemens, Munich, Germany) was carried out. By laser diffraction (Mastersizer 2000 LF from Malvern Instruments, Malvern, UK), the particle size distribution was determined. Scanning electron microscopy (SEM) images were obtained by using a Jeol JMS-6400 microscope (JEOL, Tokyo, Japan). Thermogravimetric analysis was carried out on a Mettler Toledo TGA/DTA 1 Star System (Mettler Toledo, Columbus, OH, USA). BET (Brunnauer-Emmett-Teller) [21] specific surface area was obtained by N_2_ adsorption on powder samples using a Micromeritics ASAP 2020 (Micromeritics, Norcross, GA, USA). With a Variant 1E spectrophotometer (Agilent, Santa Clara, CA, USA), the diffuse reflectance technique spectra were determined to be in the range of 300 to 900 nm, in 0.5 nm step and at a scan rate of 30 nm min^−1^.

As an application in real systems, we prepared a flooring mortar with the calcined HSL sample. This was used as an additive in the formulation of self-cleaning and de-polluting materials. To prepare the standard mortar, the formulation used was Portland BL I 52.5 N cement, the filler was composed of calcite, four different silica sands with different particle sizes, one dolomite sand, and finally, organic additives (dispersible polymer powders, plasticizer and superplasticizer). The water/cement ratio for the different formulations was adjusted to 0.45, achieving an acceptable consistency and workability according to standards [22,23]. All samples were cured at 20 °C and 65% relative humidity (RH). Compressive strength standard tests [24] were performed on prismatic specimens measuring 40 mm × 40 mm × 160 mm subjected to 28 days curing. The abrasion test was carried out with Form-Test equipment in accordance with current regulations [25]. Table S1 in Supplementary Materials shows the main characteristics of the mortar studied.

### 2.2. Self-Cleaning Test

To determine the photocatalytic capacity of the HSL samples, an organic dye was exposed to artificial sunlight (UV: 25 W/m^2^; Visible light: 550 W/m^2^,). Thus, 150 mg of waste was dispersed in 50 mL of 0.1 mM aqueous methylene blue (MB, blue dye) by continuous stirring. After light irradiation, 5 mL aliquots were taken at defined intervals of time and the remains of the catalyst were removed by centrifugation. Samples were then analyzed on a Helius Gamma spectrophotometer (Thermofisher Scientific, Waltham, MA, USA). To determine the degradability of MB, C/C_0_ was measured, where C_0_ and C are the intensities of the main absorption peak of MB (660 nm) before and after irradiation.

Another analyzed property of the mortars with HSL as a photocatalytic additive was self-cleaning; in this case the test was carried out according to standard methods [26]. The surfaces of the mortar specimens were stained with a dye solution (rhodamine-B (RhB) ethanol solution, red dye, 0.1 mM). As the standard test indicates, the samples were exposed to a solar simulator for 26 h. The color differences were analyzed over time with a Konica Minolta spectrophotometer (Tokyo, Japan), Model CM-3500d, using a 0.8 cm diameter mask. The CIE L * a * b * color space was used as a reference, although the modeling of the result was done using only the parameter a *, since when applying the RhB to the mortar, a pink color was obtained. 

The degradability of RhB is represented by the R_26_ index: [27]

Direct photolysis affects the RhB dye [28]; therefore, we calculated the R26 index taking as a reference the pink color of the samples with the HSL additive, and the white color for the standard sample.

### 2.3. NO_x_ Abatement Test

To study the photocatalytic oxidation (PCO) of NO (de-polluting action), we used a laminar flow reactor (Homemade) with a sample holder measuring 50 mm × 50 mm. A 500 mg HSL powder sample, placed in a laminar flow quartz reactor (300 cm^3^ in volume) and irradiated with artificial sunlight, was used in each photocatalytic test. The air stream was divided in two, with one part being passed through a demineralized water deposit. Then, both streams were put together to keep the relative humidity of the air stream fixed at 50 ± 10%. To carry out the NO_x_ abatement test, the desired concentration of NO (100 ppb) was obtained mixing the air and gas streams, and the new stream was sent to the photoreactor. To determine the concentration of NO, NO_x_ and NO_2_, we used a chemiluminescence analyzer (model Environment AC32M, Environment SA, Robespierre, Poissy, Cedex, France), and the final conditions were a relative humidity of 40% and a flow rate (Q) of 0.33 L/min. In order to perform the test and verify that there was no direct photolysis or adsorption on the mortars studied, the air stream and NO gas were passed over the sample in darkness, at a constant rate, for a periods of 10 min at the beginning of the test. Subsequently, the test continued illuminating the sample for 30 min. The same protocol was used to evaluate the de-polluting action of the photocatalytic mortars prepared with HSL type additives by using 50 mm × 50 mm mortar specimens. In both cases, the equation that defines the removal rate of NO is:

NO removal rate (%) = [[NO]_inlet_ − [NO]_outlet_/[NO]_inlet_] × 100

where the NO concentration is called [NO]_inlet_ in the feed stream and [NO]_outlet_ in the outlet stream.

Both the self-cleaning tests and the abatement tests of NO were carried out in triplicate, with the stated result being the average of the three measurements.

## 3. Results and Discussion

### 3.1. Waste Transformation and Characterization

The HSL samples were subjected to a two-step transformation process: milling and heating. Firstly, with the aim of enhancing heat transfer during the calcination process, the sample was milled to segregate the largest particles and to obtain a homogenised particle size. The HSL sample was subjected to a grinding treatment for 12 to 24 h. As observed in Figure S2, only the larger particles (>50 μm) could be segregated, possibly due to the intrinsic hardness of HSL. A smaller particle size was obtained after 16 h of milling; subsequently, the particles seemed to sinter into larger aggregates. The milling process had no effect on the crystallinity, nor on the phase composition of the obtained products, as shown in Figure S3. After 16 h of milling, the HSL sample was calcined at 600 °C, 750 °C and 900 °C for 4 h, being denoted as HSL600, HSL750 and HSL900 sample, respectively.

**Figure 1:**
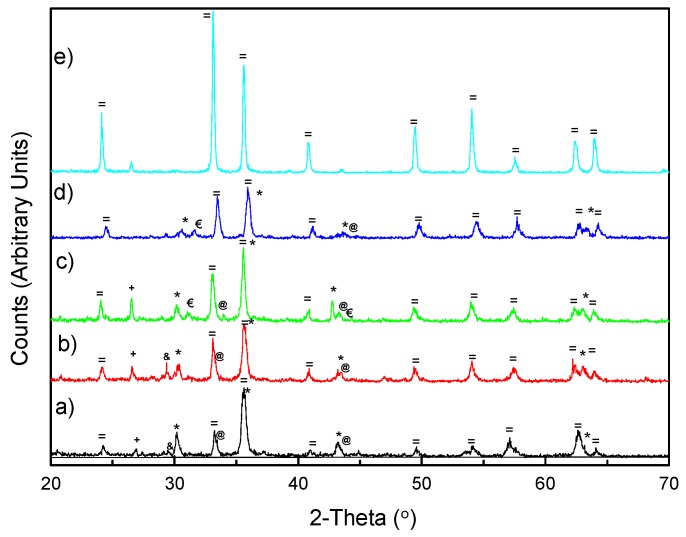
XRD patterns of (a) HSL, (b) HSL600, (c) HSL750, (d) HSL900 and (e) α-Fe_2_O_3_ samples. (+: SiO_2_; €: CaFe_2_O_4_; @: (Fe_0.6_Cr_0.4_)_2_O_3_; &: CaCO_3_;*: γ-Fe_2_O_3_/Fe_3_O_4_; =: α-Fe_2_O_3_)

The XRF chemical analysis of the HSL sample, collected after the preliminary calcination at 400 °C, shows that the main components are Fe (≈ 40%), Si (≈ 10%) Ca, Cr and Al (between 6% and 9%) and other minority elements (the total analysis shown in Table S2). This composition is in agreement with the information obtained from the corresponding XRD pattern, Figure 1a. Thus, the main crystalline phases of iron oxide found were Fe_3_O_4_/γ-Fe_2_O_3_/α-Fe_2_O_3_, and we can also see silicon oxide and calcite as sources of silicon and calcium, respectively. Other compounds such us mixed oxides (Fe_0.6_Cr_0.4_)_2_O_3_ or Ferrite (CaFe_2_O_4_) were observed. It is also interesting to note that in this residue, the only element capable of photocatalytic activity was Fe and a small amount of Ti, unlike other steel waste, mainly from slag, that also contains considerable amounts of Zn [29].

**Figure 2:**
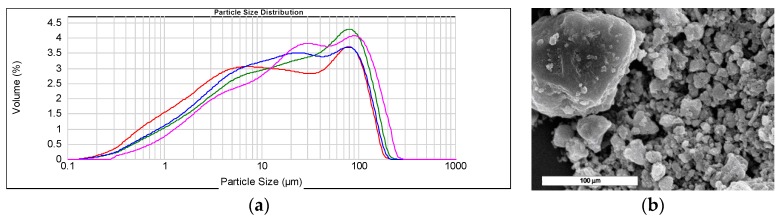
(**a**) Particle size distribution of HSL (red), HSL 600 (green), HSL 750 (blue) HSL 900 (purple) and (**b**) SEM image of HSL sample.

The analysis of the particle size of HSL shows an asymmetrical bimodal distribution, with particles in the 0.2–140 μm size range, Figure 2a. The majority of the particles displayed particle sizes lower than 20 μm. Another significant number of particles exhibited sizes between 30 and 150 μm. This distribution was confirmed by electron microscopy. The particles corresponding to both fractions of size can be observed in Figure 2b.

In the case of iron oxides, better photocatalytic activity is expected for the hematite phase, α-Fe_2_O_3_, because of its inherent semiconducting properties. Therefore, it is of interest to increase the available amount of α-Fe_2_O_3_ in HSL. As observed from the XRD pattern (Figure 1), in all cases, there was an increase in the amount of hematite phase when the temperature was increased. Also, we observed the relationship between the main reflection for different iron oxide (*) and α-Fe_2_O_3_ (=), and the increase with the treatment temperature ( S ). As predicted from TGA analysis (Figure S1), the peak at 29.2 in 2θ, corresponding to calcite phase, decreased when the temperature increased and disappeared for T ≥ 750 °C. Concerning the period of calcination, increasing the heating time to 8 and 12 h showed no significant differences between the representative phases identified by X-ray diffraction (Figure S4). In all cases, the conversion to the hematite phase is very similar, so the optimal calcination time can be established in 4 h.

**Figure 3:**
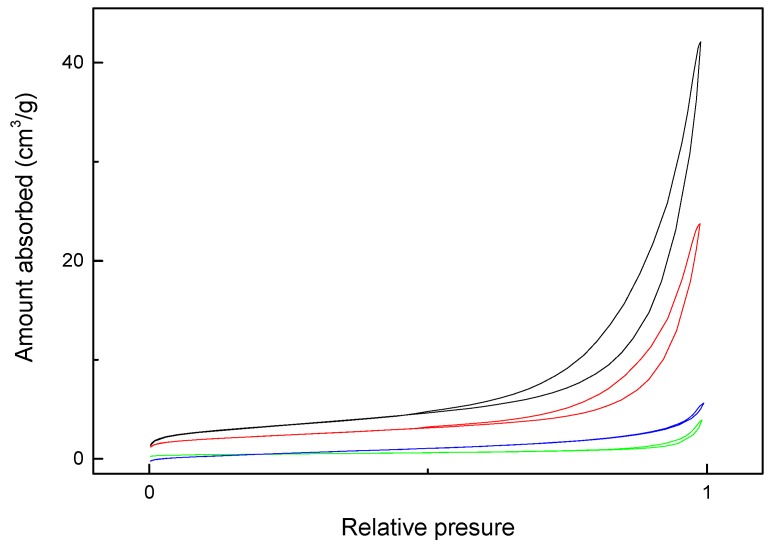
Nitrogen adsorption-desorption isotherms of HSL600 (black), HSL750 (red), HSL900 (green) and hematite (blue) samples.

Because both self-cleaning and self-depolluting properties originate from the heterogeneous photochemical reaction that takes place on the surface of the photocatalyst, it is of interest to know about its porous microstructure and optical properties. In this sense, the main data about the porous microstructure are shown in Table 1, and data obtained from the corresponding N_2_ adsorption-desorption isotherms are shown in Figure 3. The isotherm curves, classified as type III [30], exhibited a H3 hysteresis loop characteristic of aggregates with loose assemblage. It is worth noting that the BET surface, pore volume and average pore size values decreased as the calcination temperature increased. Thus, the BET area of the HSL900 sample is an order of magnitude lower than that of the HSL600 sample. Thus, by increasing the calcination temperature, the microstructure becomes less porous, hindering the accessibility to the reactant molecules, precluding the use of this sample as a photocatalyst, as was subsequently demonstrated.

**Figure 4:**
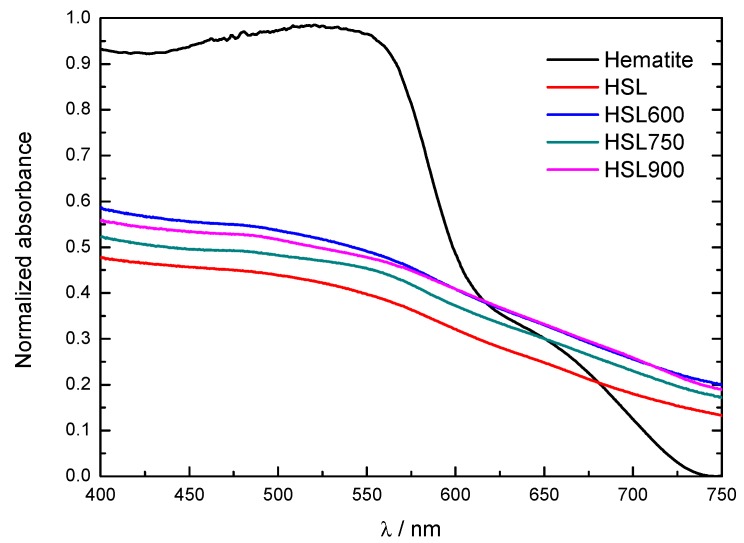
The absorption spectra for HSL type and Fe_2_O_3_ samples.

Using UV-visible diffuse reflectance spectroscopy, we studied the optical properties of the samples. The absorption spectra revealed that HSL samples shared a similar adsorption edge, see Figure 4. The HSL-type samples exhibited a broad band located at approximately 550 nm, similar to the maximum observed for the hematite pattern. The transformed waste shows a lower adsorption level than hematite, probably because it consists of a mixture of mineralogical compounds differing in their optical adsorption abilities. The adsorption intensity increased from HSL to HSL600 and HSL900 samples, which is in agreement with the enrichment in α-Fe_2_O_3_ observed after the heating process.

#### 3.1.1. Self-Cleaning and Self-Depolluting Properties

**Figure 5:**
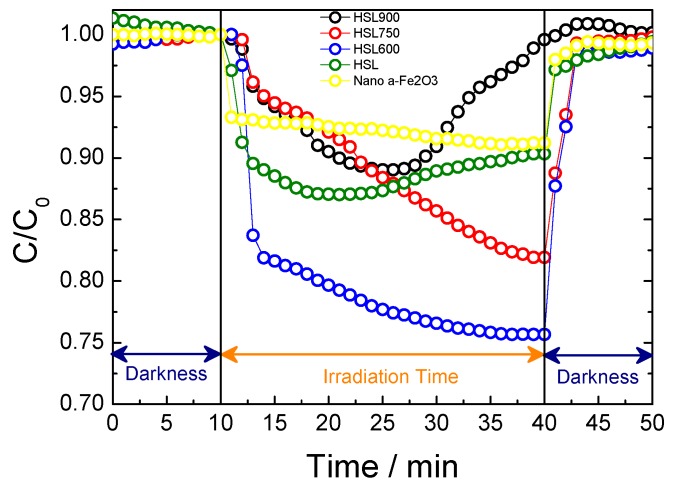
NO gas concentration profile evolution measured during the photochemical test, for α-Fe_2_O_3_ pattern and HSL samples.

The ability of HSL-type samples to self-clean and self-depollute, as induced by photochemical reactions, was analysed. Firstly, in the HSL samples, the model employed to analyse the decontamination properties was the determination of the photocatalytic activity of the NO gas exposed to the radiation. The concentration of NO versus time (dark periods and irradiation) for the HSL samples was determined and compared with the standard of α-Fe_2_O_3_, as can be seen in Figure 5 [31]. Regarding the NO concentration profiles over time, the amount of NO removed increased when the BET surface of the photocatalyst increased. Only in the case of samples HSL900, the process of elimination of NO seems limited in time, due to the small number of active sites (1.6 m^2^·g^−1^). The concentration of NO only underwent a sudden decrease under irradiation, meaning that once the activation of the α-Fe_2_O_3_ sites occurs, the oxidation of the pollutant took place by heterogeneous photocatalytic reaction [15,32]. The increase of the specific surface area, for each photocatalyst, causes an increase in the NO photochemical oxidation reaction. Thus, because of its negligible BET surface, the photochemical process hardly accounts for sample HSL900. The best removal rate, around 25%, was obtained for the HSL600 sample, being superior to that exhibited by the α-Fe_2_O_3_ (BET area = 9.14 m^2^·g^−1^ and Pore size = 7.53 nm [31]; NO removal = 10% [15]) standard and other hematite based De-NOx photocatalyst.

On the other hand, the model used to study the self-cleaning property was the photochemical degradation of methylene blue (MB) exposed to solar radiation (Figure S5). This experiment was limited to the HSL600 sample, once the De-NO_x_ test clearly showed its best photocatalytic activity in comparison with the other HSL type samples.

**Figure 6:**
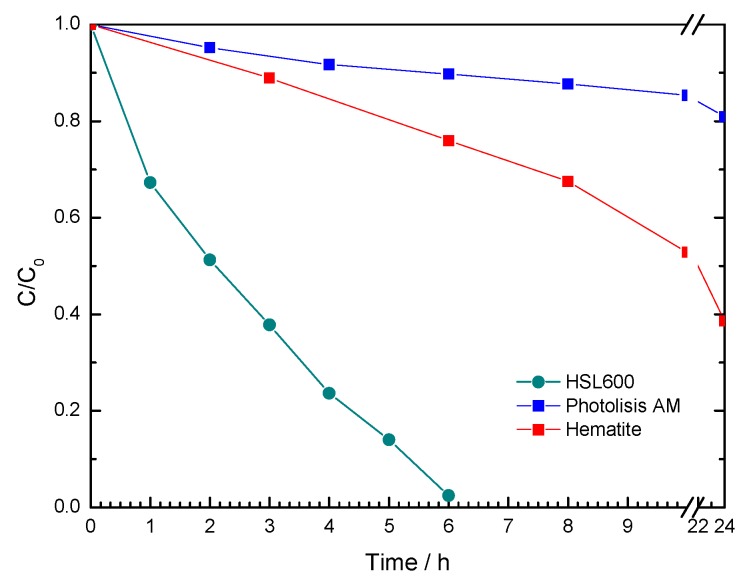
Methylene Blue photochemical degradation by photolysis, α-Fe_2_O_3_ and HSL600 samples.

As observed in Figure 6, the MB concentration decreases with irradiation time in a more favourable way for the HSL600 sample compared to the α-Fe_2_O_3_ standard. The observed behaviour could be understood considering that iron phases can cause a photocatalytic process. Thus, these phases lead to water decomposition and molecular oxygen reduction, generating OH• and ^•^O^2−^ radicals, respectively. These species can mineralize the organic compounds due to the fact they are good reactive oxidizing agents [33]. Additionally, this behaviour is consistent with the microstructure data obtained for the HSL600 sample. Apart from its BET surface area, the biggest pore size facilitates the adsorption of the Methylene Blue large molecules in the HSL sample, improving the efficiency in the photochemical degradation [34].

Therefore, as previously reported for iron oxides, the ability to promote the decomposition of dyes [35,36] and NO_x_ gases [37,38] was stated for HSL type photocatalysts. Because of their iron oxide content, when Fe_2_O_3_ particles are irradiated by solar light, photogenerated electrons (e^−^) pass from the valence band to the conduction band, while in the valence band, the holes (h^+^) are photogenerated simultaneously, as shown in equation [39].

(1) $${\mathrm{Fe}}_{2}O_{3}\overset{h\nu }{\to }{\mathrm{Fe}}_{2}O_{3}\left(h^{+}\right)+{\mathrm{Fe}}_{2}O_{3}\left(e^{-}\right)$$

On the Fe_2_O_3_ surface, holes are photogenerated, which then migrate and produce hydroxyl radicals when they react with water molecules, as shown in Equation (2).

(2) $${\mathrm{Fe}}_{2}O_{3}\left(h^{+}\right)+H_{2}O\to {\mathrm{Fe}}_{2}O_{3}+H^{+}+{}^{•}\mathrm{OH}$$

(3) $${\mathrm{Fe}}_{2}O_{3}\left(e^{-}\right)+O_{2}\to {\mathrm{Fe}}_{2}O_{3}+{}^{•}O_{2}^{-}$$

(4) $${}^{•}O_{2}^{-}+H^{+}\to {}^{•}{\mathrm{HO}}_{2}$$

The radicals ^•^OH, ^•^HO_2_ and ^•^O_2_ are responsible for the degradation of dyes and NO_x_ gases.

#### 3.1.2. Self-Cleaning and Self-Depolluting Building Materials

With the aim of applying these photocatalysts to real systems, we studied the addition of the HSL600 sample in a flooring mortar, as an example of a new photocatalytic building material. Previous studies [11,40] have shown how the internal microstructure affects the mechanical and photocatalytic properties of cement-based mortars Taking this fact into consideration, and with the objective of checking the behaviour of the mortar with HSL600, we examined different mortar formulations, changing HSL with sands and keeping the percentage of cement and additives constant. (Supplementary Materials, Table S1) In our case, according to the particle size distribution, we replaced the CaCO_3_ filler with 5% and 10% HSL. Thus, the samples corresponding to the new formulation mortars were named M5-HSL600 and M10-HSL600, with the initial number being the amount of substituent HSL600 in mortar. Concerning the physical and mechanical properties, the values of flexural, compression and abrasion resistances (Supplementary Materials, Table S4) were obtained after standard curing. In all cases, the values were higher than 7 N/mm^2^ (flexural strength), 30 N/mm^2^ (compressive strength) and lower than 400 mm^3^ (abrasion resistance). Optimal values were deduced from the experience of the application of these kinds of mortars [40].

**Figure 7:**
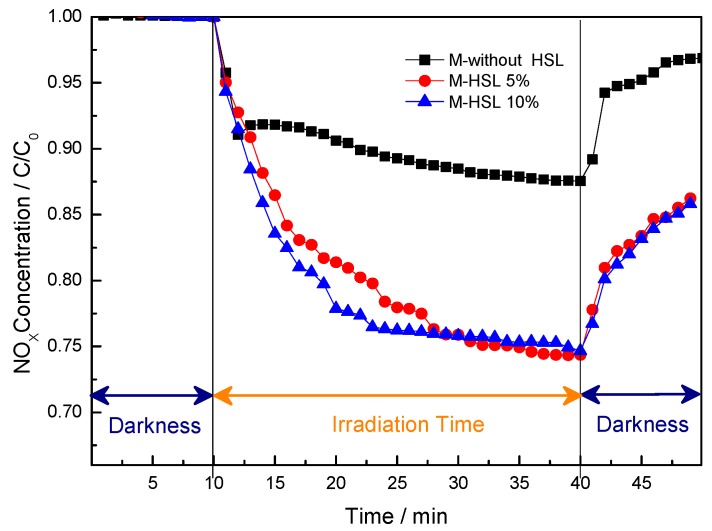
NO gas concentration profile evolution measured during the photochemical test, in flooring mortar samples.

Subsequently, the potential applications of new flooring photocatalytic mortars were examined. Photocatalytic flooring mortar can reduce urban pollution through photocatalysis and by enhancing the aesthetic durability of cement-based materials. In accordance with this, Ai et al. [41] confirmed that the removal of NO accounts for its PCO to NO_2_^−^/NO_3_^−^. In this study, the presence of the HSL 600 sample in the mortar formulation clearly enhanced the PCO removal of NO, Figure 7. The removal of NO was slightly enhanced by increasing the addition of HSL600 from 5% to 10%).

**Figure 8:**
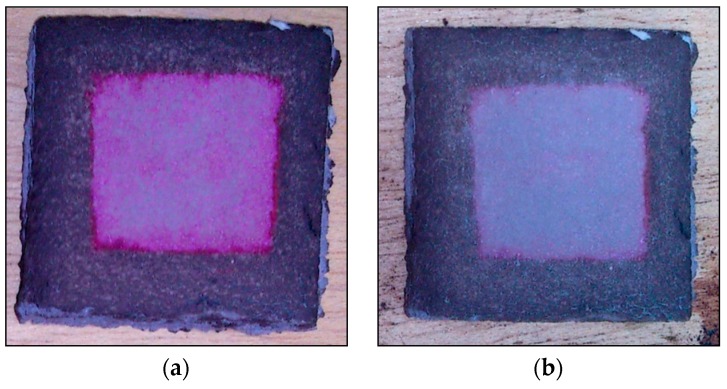
The RhB degradation test. Mortar specimen before (**a**,**b**) after 26 h of light irradiation.

Finally, according to the standard for building materials, we used a RhB degradation test to check the ability of the photocatalytic flooring mortars to self-clean. Firstly, in order to carry out the test, small pieces (4 cm × 4 cm) of mortar were protected with waterproofing material on their external surface. Subsequently, the mortar was impregnated in the center without waterproof material, with 1.5 mL of a RhB ethanol solution (100 mg/L, 1.5 g), as shown in Figure 8. When the dye was dry, light irradiation was started.

The red stain was reduced after irradiation. The mortar without waste in its composition remained unchanged (image not shown). The R_26_ index value is a quantitative evaluation of RhB dye photodegradation. In samples M5-HSL600 and M10-HSL600, the R_26_ values were 21.7% and 56.55%, respectively. Therefore, the results obtained for M10-HSL600, with dye degradation in the mortar higher than 50%, are indicative of a positive self-cleaning performance.

## 4. Conclusions

The main conclusions from this work are the following:

- The appropriate management of HSL waste could transform this waste into a new material with high added value as a sustainable photocatalytic material.
- HSL waste is iron rich, its phases are mainly hematite (α-Fe_2_O_3_), mixed oxides (Fe_0.6_Cr_0.4_)_2_O_3_ or Ferrites (CaFe_2_O_4_).
- It is necessary to perform thermal pretreatment in order to remove traces of impurities.
- The increased amount of α-Fe_2_O_3_ phase is obtained in calcinated samples at temperatures higher than 600 °C.
- The calcination treatment affects the pore microstructure of the sample, decreasing porosity and surface area in samples calcinated at higher temperatures in the 600–900 °C range.
- The specific surface area was found to be an important experimental parameter. A decrease in the specific surface of the treated waste leads to worse photocatalytic performance.
- Discoloration of dyes and NO removal from air were positively tested with the HSL powders and mortars, indicating their ability to act as photocatalytic materials.

The proper treatment of the HSL waste and it is subsequent use, combined with atmospheric oxygen, taking advantage of sunlight and water present in the form of humidity, allows depollution of the air and a self cleaning effect of building facades to be obtained simultaneously.

## Figures and Tables

**Table 1:** Surface area and porosity parameters for the different samples.

Sample	BET Surface Area (m^2^·g^−1^)	Pore Volume (cm^3^∙g^−1^)	Average Pore Size (nm)
HSL600	11.48 ± 0.05	0.065	22.695
HSL750	7.96 ± 0.05	0.036	18.467
HSL900	1.61 ± 0.02	0.006	15.029
Hematite	5.10 ± 0.08	0.017	7.524

## Supplementary Materials

The following are available online at http://www.mdpi.com/1996-1944/12/4/621/s1, Table S1: Mortar formulation studied; Table S2: XRF data of the HSL waste after previous treatment expressed as simple oxides. (%); Table S3: Relationship between the main reflection for different iron oxide (*) and α-Fe_2_O_3_ (=); Table S4: Flexural, compression strength and abrasion resistance after 28 days for Reference mortar (M) and mortar with different percents of HSL replacement (5% and 10%); Figure S1: Thermogravimetric analysis of the waste as received; Figure S2: Particle size distribution of HSL waste after grinding for 12 h (red), 16 h (green), 20 h (blue) and 24 h (pink); Figure S3: X-Ray diffraction data for HSL samples (a) as received, (b) after grinding for 12 h and (c) after grinding for 24 h. (SiO_2_ +; (Fe_0.6_Cr_0.4_)_2_O_3_ @; CaCO_3_ &; γ-Fe_2_O_3_/Fe_3_O_4_ *; α-Fe_2_O_3_ =); Figure S4: XRD data for HSL waste (black), after 600 °C 4 h (red), 8 h (blue) and 12 h (green). (SiO_2_ +; (Fe_0.6_Cr_0.4_)_2_O_3_ @; CaCO_3_ &; γ-Fe_2_O_3_/Fe_3_O_4_ *; α-Fe_2_O_3_ =); Figure S5: Evolution of the absorption spectrum of methylene blue over time.
