# Supplementary material for: Use of Steel Industry Wastes for the Preparation of Self-Cleaning Mortars
Source: Materials (Basel). 2019 Feb 19;12(4):621. doi: 10.3390/ma12040621 (PMC6416718; doi:10.3390/ma12040621)
Supplement: Supplementary file 1 [file materials-12-00621-s001.pdf]

Supplementary Materials: Use of Steel Industry Wastes for the Preparation of Self-Cleaning Mortars

José Balbuena, Luis Sánchez and Manuel Cruz-Yusta *

Table S1. Mortar formulation studied.

Mortar Components	M-1	M-2	M-3	M-4	M-5	M-6	M-7	M-8
Sand 1 (400–3000 µm)	20.23	10.23	20.23	-	-	12.23	22.23	43.47
Sand 2 (300–3000 µm)	-	5	-	22.23	20.23	5	-	-
Sand 3 (200–1500 µm)	35.47	40.47	20	33.47	15	15	-	12.23
Sand 4 (150–800 µm)	-	-	-	-	20.47	23.47	33.47	-
Sand 5 (100–500 µm)	-	-	20.47	-	-	-	-	-
Filler	10	10	10	10	10	10	10	10
Cement	33	33	33	33	33	33	33	33
Redispersible polymer	1	1	1	1	1	1	1	1
Surfactant wetting	0.05	0.05	0.05	0.05	0.05	0.05	0.05	0.05
Fluidifying	0.25	0.25	0.25	0.25	0.25	0.25	0.25	0.25

Table S2. XRF data of the HSL waste after previous treatment expressed as simple oxides. (%).

	Fe_2O_3	SiO_2	CaO	Cr	Al_2O_3	MgO	Ni	MnO
**HSL Waste**	41.94	10.64	8.93	7.56	6.75	4.14	1.95	0.98
	TiO_2	P_2O_5	Cu	Na_2O	K_2O	Zn	L.O.I	TOTAL
	0.32	0.28	0.23	0.22	0.21	0.16	11.57	95.87

Table S3. Relationship between the main reflection for different iron oxide (*) and α-Fe_2O_3 (=).

Sample	Relationship
HSL	1
HSL 600	≈ 0.36
HSL 750	≈ 0.18
HSL 900	≈ 0.14

Table S4. Flexural, compression strength and abrasion resistance after 28 days for Reference mortar (M) and mortar with different percents of HSL replacement (5% and 10%).

Mortar	Flexural Strength 28 d/$N \cdot mm^{-2}$	Compression Strength 28 d/$N \cdot mm^{-2}$	Abrasion Strength 28 d/mm^3
M	9.6	76.4	89.0
M5HSL	10.3	67.1	123.0
M10HSL	10.0	62.0	147.0

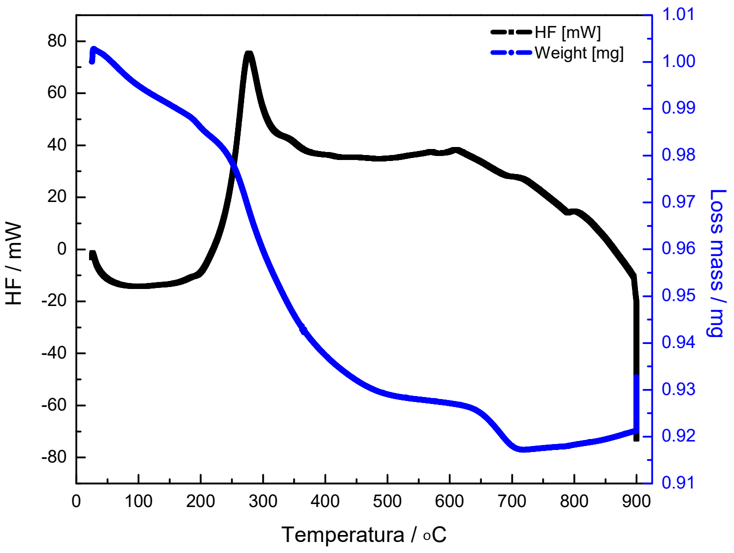

Figure S1. Thermogravimetric analysis of the waste as received.

The thermogravimetric analysis of the samples as received shows around of 7% of the weight sample, lost after calcination to 400 degrees. This loss is associated to the organic matter.

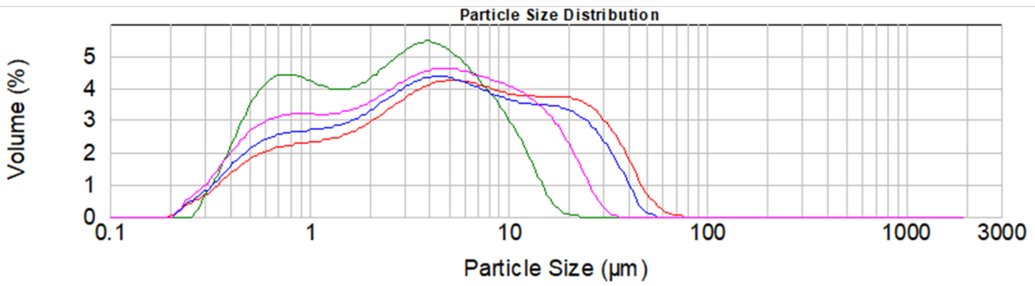

Figure S2. Particle size distribution of HSL waste after grinding for 12 h (red), 16 h (green), 20 h (blue) and 24 h (pink).

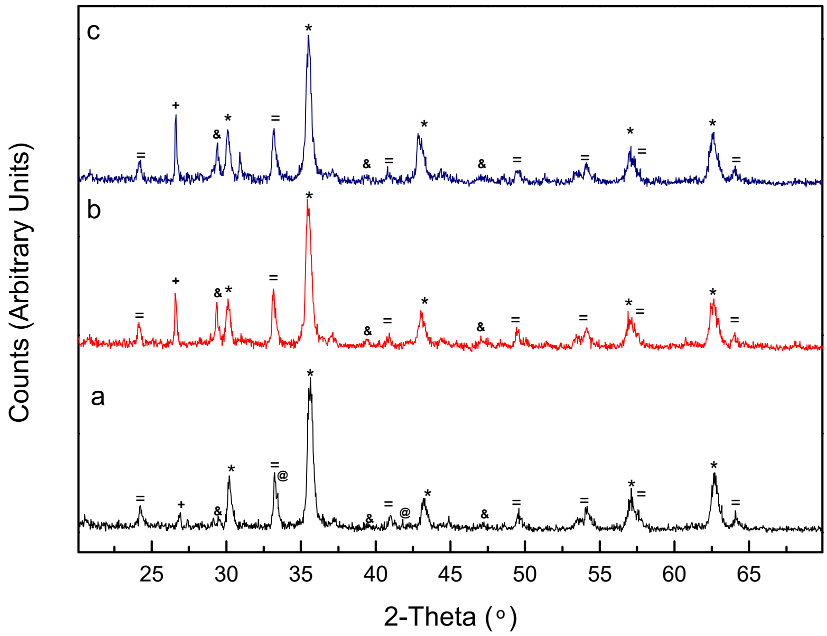

Figure S3. X-Ray diffraction data for HSL samples (**a**) as received, (**b**) after grinding for 12 h and (**c**) after grinding for 24 h. (SiO_2 +; $(Fe_{0.6}Cr_{0.4})_2O_3$ @; $CaCO_3$ &; γ-Fe_2O_3/Fe_3O_4 *; α-Fe_2O_3 =).

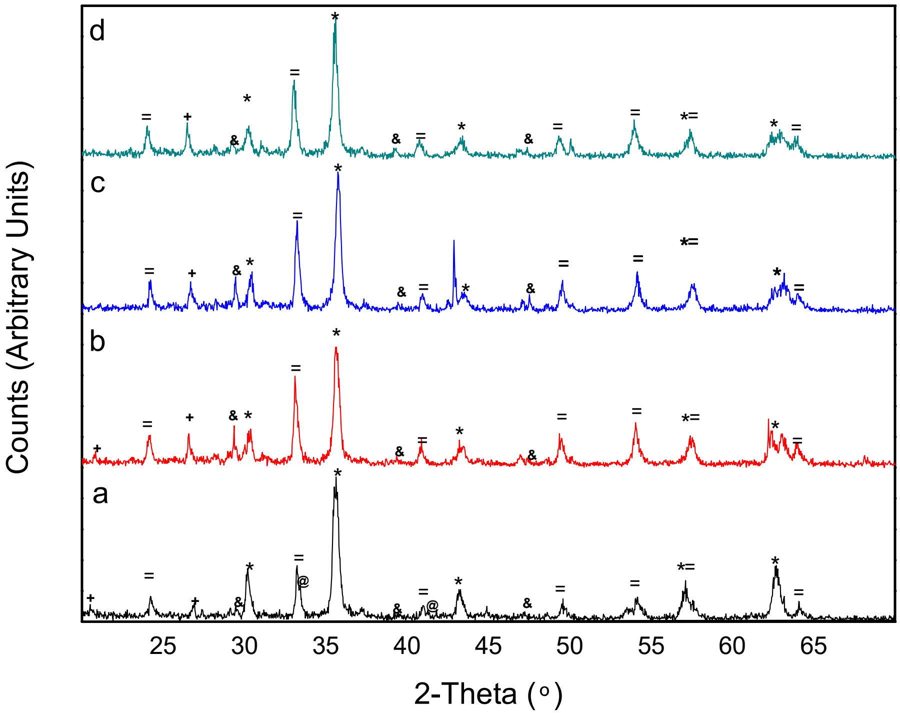

Figure S4. XRD data for HSL waste (black), after 600 °C 4 h (red), 8 h (blue) and 12 h (green). (SiO_2 +; $(Fe_{0.6}Cr_{0.4})_2O_3$ @; $CaCO_3$ &; γ-Fe_2O_3/Fe_3O_4 *; α-Fe_2O_3 =).

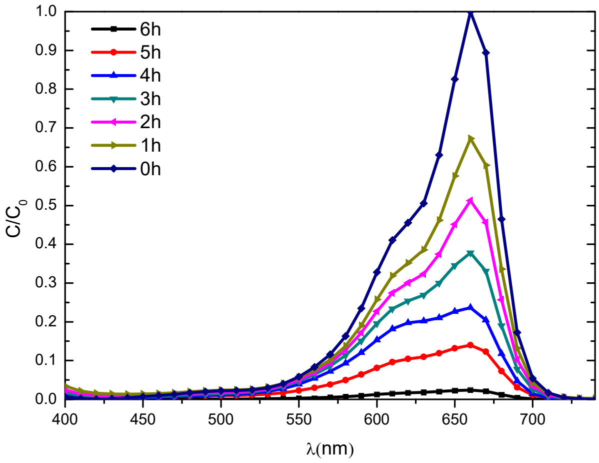

Figure S5. Evolution of the absorption spectrum of methylene blue over time.
